# Laser Power-Dependent Microstructural Evolution and Fracture Mechanisms in Ti80 Titanium Alloy Welds: A Multi-Scale Investigation

**DOI:** 10.3390/ma19010116

**Published:** 2025-12-29

**Authors:** Chuanbo Zheng, Zhanwen Yang, Guo Yi, Liuyu Zhang, Xiaomeng Zhou, Xinyu Yao

**Affiliations:** 1 School of Metallurgy and Engineering, Jiangsu University of Science and Technology, Zhangjiagang 215600, China; 15952802516@139.com; 2 Suzhou Sitri Welding Technology Research Institute Co., Ltd., Zhangjiagang 215600, China

**Keywords:** Ti80 titanium alloy, laser welding, mechanical properties, simulation

## Abstract

The laser welding of 4 mm thick Ti80 alloy under different powers was analyzed, and the weld morphology, microstructure, and mechanical properties were studied. A simulation model was established based on ABAQUS, and laser welding simulations were conducted using 2520 W and 3000 W laser welding power sources to analyze the temperature field and stress field, which were verified by experiments. The increase in power changed the weld morphology from Y-shaped to X-shaped and affected the number of pores in incomplete and complete penetration. The microstructure in the weld zone presented fine acicular α′ phase. Subsequently, grain boundary distribution maps, Kernel Average Misorientation (KAM) maps, and geometrically necessary dislocation (GND) density maps were generated through electron backscatter diffraction (EBSD) analysis. These comprehensive data visualizations enabled multi-dimensional investigation, establishing and analyzing correlations between laser welding parameters, microstructural evolution, and mechanical properties in Ti80 titanium laser welding. The hardness of the base material was 320 HV to 360 HV, and it increased from 420 HV to 460 HV in the weld zone. At 3000 W, the tensile strength reached 903.12 MPa, and the elongation was 10.40%, indicating ductile fracture. The simulation results accurately predicted the maximum longitudinal residual stress in the weld zone, with an error of 1.65% to 1.81% of the measured value.

## 1. Introduction

In the 21st century, the ocean has emerged as a critical domain of competition among nations in political, economic, and military spheres. Research into marine science and the development of marine engineering technologies have been elevated to the strategic decision-making level of national policies. Titanium not only possesses high strength and excellent corrosion resistance but also offers a significant lightweight advantage that is absent in other commonly used marine engineering materials. Replacing traditional materials such as stainless steel and aluminum with titanium can substantially extend the service life of marine engineering equipment, reduce maintenance and repair costs, and enhance the safety and reliability of marine operations [1,2,3]. Furthermore, the corrosion resistance of titanium alloys in marine environments can be significantly enhanced by fabricating surface coatings with superhydrophobic properties [4].

TC4, Ti31, and Ti80 are among the most commonly used titanium alloys in marine engineering. Among these, Ti80, a near-α type titanium alloy, exhibits excellent mechanical properties and superior weldability [5]. Consequently, it finds extensive application in critical components such as large-diameter pressure pipelines, high-pressure vessels, deep-sea submersibles, ship pressure hulls, and welded structural components of vessels. Given its widespread use in significant marine engineering projects, conducting research on the weldability of Ti80 titanium alloy is of paramount importance.

Currently, research on the welding of Ti80 titanium alloy primarily focuses on friction stir welding (FSW) of T-joints [6], electron beam welding (EBW) under constrained conditions [7], and laser melting welding of dissimilar joints [8]. However, relatively limited research has been conducted on laser self-melting welding of Ti80 titanium alloy. Friction stir welding is a solid-state welding method that offers advantages such as no pollution, no smoke or dust, no radiation, and environmental safety. Nevertheless, in marine applications, especially for precision structural components and critical structures like submarine hulls, FSW results in a relatively low depth-to-width ratio and lower connection strength, making it potentially unsuitable for these applications. Electron beam welding requires operation in a vacuum environment, imposing stringent requirements on the working conditions. Laser welding, on the other hand, boasts high efficiency, high energy density, excellent joint quality, and superior work efficiency [9,10,11]. In this study, 4 mm-thick Ti80 titanium alloy plates were subjected to fiber laser welding to investigate their microstructure and mechanical properties. Additionally, an ABAQUS-based simulation model was established to predict and analyze the temperature and stress fields during and after the laser welding process. The accuracy of the simulation model was subsequently validated through experimental testing.

## 2. Experiment and Simulation Modeling Methods

### 2.1. Materials and Welding

**Figure 1:**
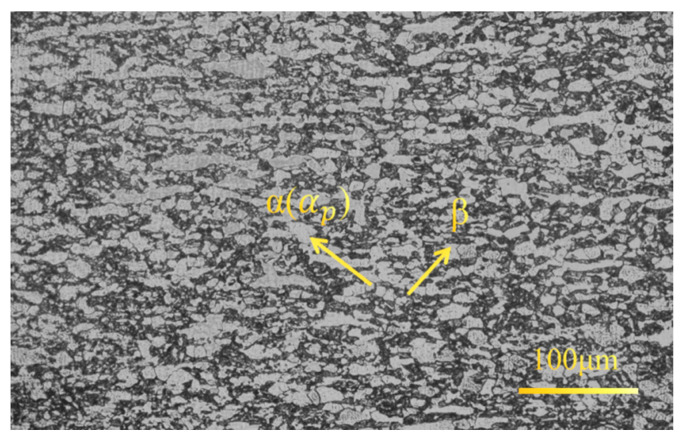
Metallographic photographs of the Ti80 titanium alloy plate.

This BM (base material) is Ti80 titanium alloy that has undergone forging and is in the annealed plate. As shown in Figure 1, its microstructure is composed of a considerable amount of primary α($\alpha_{p})$ and a small portion of β. Furthermore, a large number of secondary α($\alpha_{s})$ are distributed within the β. It is a near-α type titanium alloy. The chemical composition of the Ti80 is shown in Table 1.

**Figure 2:**
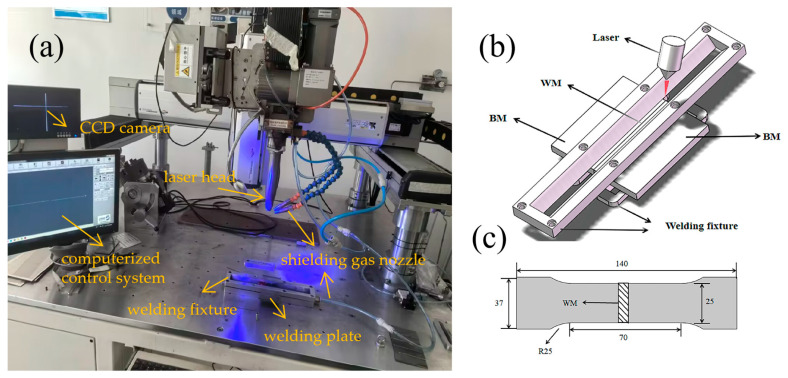
Fiber laser welding system: (**a**) laser welding platform; (**b**) welding fixture; (**c**) tensile specimen dimensions.

The plate was cut into multiple small plates, each with a length of 150 mm, a width of 75 mm, and a thickness of 4 mm. Firstly, sand the weld area using sandpapers with varying grit sizes. Secondly, treat the stains and oxide layer on its surface with Kroll reagent (2% HF + 4% HNO_3_ + 94% H_2_O) (Hengguangfan Scientific Experiment Station, Sanming, China), and finally clean it with absolute ethanol (Wuxi Zhanwang Chemical Reagent Co., Ltd, Wuxi, China). These treated plates will subsequently be grouped in pairs and placed onto the laser welding platform for laser welding. The model of the adopted laser is YLLM-W-M-5015-A (GW LaserTech LLC, Shanghai, China), and that of the welding head is UW-30P-90180C-WDZ-6KW (UW Laser Co., Ltd, Shenzhen, China). In this experiment, argon gas (Ar) (Air Liquide (Changshu) Gas Co., Ltd, Changshu, China) with a purity of 99.99% was used as the shielding gas, and the gas flow rate was set at 25 L/min. Given that the fiber laser utilized was a multimode laser, its defocus tolerance was approximately ±0.2 mm. The schematic diagrams of the fiber laser welding system and the tensile test specimen are presented in Figure 2.

The specific parameters related to welding are presented in Table 2 as follows. In Table 2, the heat input is jointly determined by the laser power and the welding speed. The relevant formula is presented as follows:

E = P/V(1)

where E is heat input, P is laser power, V is welding speed.

### 2.2. The Vickers Hardness Test and the Residual Stress Test

**Figure 3:**
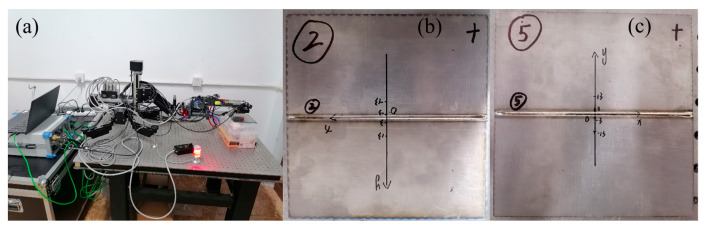
XRD Residual Stress Testing System: (**a**) DS-21P; (**b**) specimen 2; (**c**) specimen 5.

This specimen utilized for tensile testing adheres to Standard ISO 4136:2022 [12], and the specimens employed for residual stress testing adhere to Standard ISO 17636-1:2013 [13]. In this research, the Vickers hardness tester utilized was HVS-50 (Shanghai Qinming Optical Instrument Co., Ltd., Shanghai, China). The indentation force was 9.807 N, and the loading time was 15 s. Figure 3 shows the XRD residual stress measurement system used: the DS-21P Portable Residual Stress Measurement Device (Dandong Haoyuan Instrument Co., Ltd, Dandong, China), Axio Scope.A1 optical microscope (OM) (Carl Zeiss AG, Oberkochen, Germany), Stemi 305 Stereoscopic Microscope (STM) (Carl Zeiss AG, Oberkochen, Germany), Symmetry S3 Electron Back Scatter Diffraction (EBSD) (Oxford Instruments Technology (Shanghai) Co., Ltd, Shanghai China) and JSM-6510LA scanning electron microscope (SEM) (JEOL Ltd, Tokyo, Japan) were used to observe and analyze the cross-sectional morphology and microstructure of welded joints and tensile specimen sections.

### 2.3. Simulation Methods

To investigate the relationship between thermal cycling and microstructural evolution in laser-welded joints, this study established a numerical heat source model using ABAQUS 2022 and further analyzed the temperature and stress distributions within the weld. This simulation adopts two laser power parameters of (a) 2520 W and (b) 3000 W as the power of the simulation heat source.

**Figure 4:**
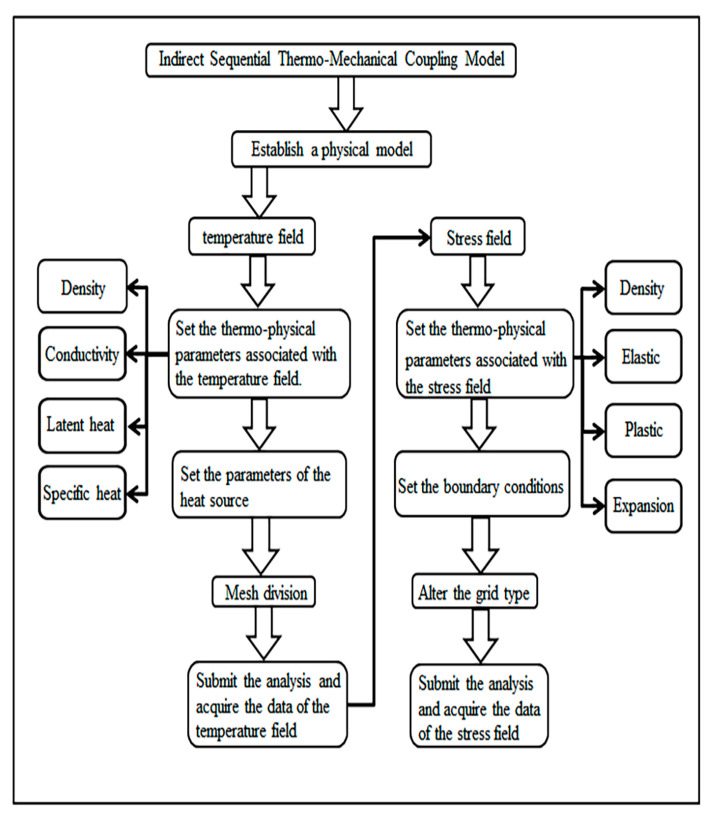
Schematic illustration of the main procedures in indirect thermal sequential coupling.

To minimize the computational time cost to the greatest extent, the simulation model in this study employed an indirect sequential thermo-mechanical coupling approach. The temperature field was first established and analyzed to obtain the necessary data, which was then imported into the stress field. The specific process is illustrated in Figure 4.

**Figure 5:**
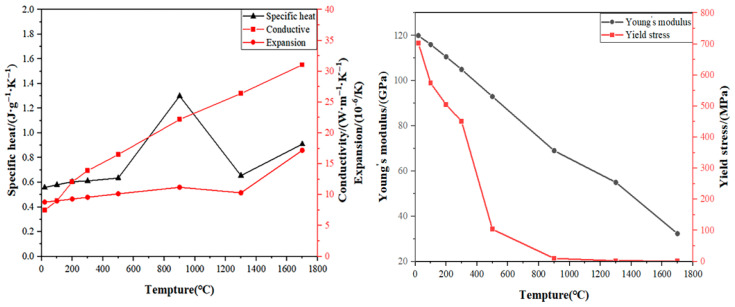
The thermal physical property parameters of Ti80 titanium alloy.

By consulting the relevant literature [14], the thermal physical property parameters of the materials required for Ti80 in the simulation were established. Given the computational cost and model complexity, the effects of martensitic phase transformation in titanium alloys were not incorporated into this simulation. Table 3 lists the inherent properties of the Ti80 titanium alloy. Figure 5 exhibits the relevant thermophysical properties of the Ti80 titanium alloy.

**Figure 6:**
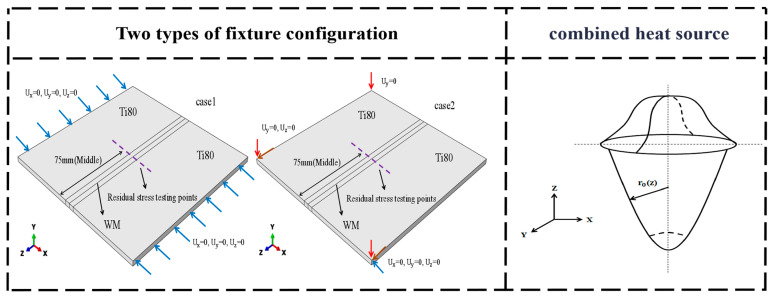
Fixture configuration and schematic illustration of combined heat source.

The surface convective heat transfer coefficient is prescribed as 40 J/$m^{2}$, and the surface radiation emissivity is set at 0.7 [15]. Since the welding is conducted under room temperature conditions, the initial temperature is designated as 20 °C. The titanium alloy model was discretized into 48,000 meshes. In the temperature field, the mesh type is designated as DC3D8, whereas in the stress field, it is specified as C3D8R. To reduce the complexity of the model and minimize time costs, the fixture configuration of the laser welding model was simplified. Two clamping methods were employed in the boundary conditions to investigate their effects on residual stresses, including the conventional fixture setting case1 and the ideal three-point fixture setting case2. The specific configuration is shown in Figure 6.

In the case of complete penetration in laser welding, two classical heat source model combinations are widely used: (1) the combination of a double ellipsoidal surface heat source and a conical heat source [16], and (2) the combination of a Gaussian surface heat source and a conical heat source [17]. Both approaches are capable of effectively simulating the attenuation of thermal energy along the material thickness direction in deep penetration welding. Among the two, the double ellipsoidal surface heat source combined with a conical heat source offers higher simulation accuracy. However, this model involves a larger number of calibration parameters and demands greater computational resources. The utilization of combined heat sources with power distribution capabilities enhances simulation accuracy. Given the constraints on experimental costs, this study employs Gaussian-distributed surface heat sources and conical heat sources characterized by increasing peak heat flux and depth-dependent radius for simulation. The overall effective heat flux density is Q is as follows:

(2) $$Q=\mu_{1}q_{1}+\mu_{2}q_{2}$$

The equation of the Gaussian surface heat flux density $q_{1}$ is

(3) $${\;q}_{1}=\frac{3P_{1}}{{\pi R}_{0}^{2}}\cdot \exp(-\frac{3r^{2}}{R_{0}^{2}})$$

The equation of the Conical heat flux density q_2_ is

(4) $$q_{2}=\frac{9e^{3}P_{2}}{\pi \left(e^{3}-1\right)}\cdot \frac{\exp\left(-\frac{3r^{2}}{r_{0}^{2}}\right)\;}{\left(Z_{1}-Z_{2}\right)\left(r_{1}^{2}+r_{1}r_{2}+r_{2}^{2}\right)}$$

In the above equation, $\mu_{1}$ and $\mu_{2}$ are heat distribution coefficients, $P_{1}$ and $P_{2}$ are the effective power of the heat source, $R_{0}$ is the effective action radius of the heat source, r represents the distance to the center of the Gaussian heat source, $Z_{1}$ and $Z_{2}$ denote the thickness-directional coordinates of the upper and lower surfaces of the inverted cone, r_1_ and r_2_, respectively, denote the effective heating radii of the upper and lower surfaces of the inverted cone, and r_0_ represents the heating radius value that decays along the thickness direction.

## 3. Results and Discussion

### 3.1. Effect of Laser Power on Weld Morphology

**Figure 7:**
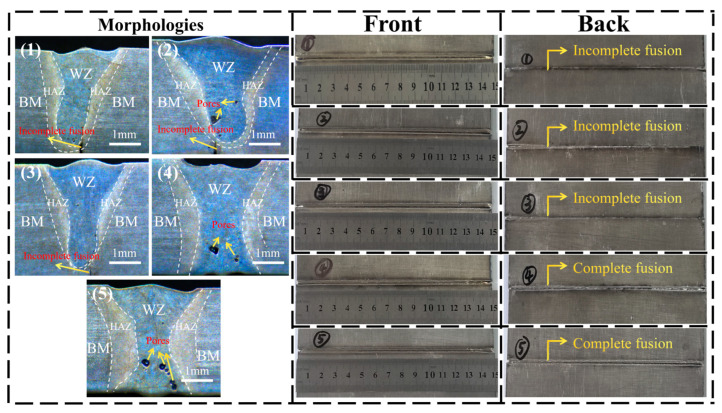
Effect of laser power on the surface morphologies of the front and back surfaces of the weld seam: (1) 2400 W, (2) 2520 W, (3) 2640 W, (4) 2760 W, and (5) 3000 W.

The impact of laser power on the surface topography of the front and back sides of the weld seam is shown in Figure 7.

As shown in Figure 7, the surface morphology of the weld seam is satisfactory, with no evident spatter or porosity. The frontal weld seam exhibits a silver-white or slightly yellow coloration, indicative of superior weld quality. However, on the reverse side, samples 1, 2, and 3 demonstrate incomplete penetration due to inadequate laser power, whereas the back welds of samples 4 and 5 exhibit favorable formation.

With the increase in laser power, the cross-sectional morphology of the weld seam gradually transforms from a Y-shaped one to an X-shaped one. The portion of the weld seam that is not fully penetrated gradually reduces until complete penetration is achieved.

**Figure 8:**
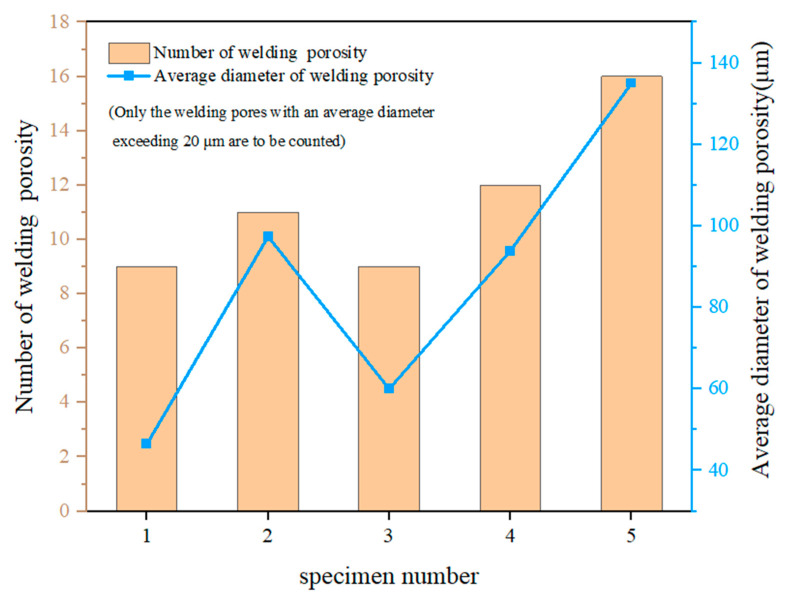
The number of pores and the average diameter of pores in the cross-sections of different specimens: (1) 2400 W, (2) 2520 W, (3) 2640 W, (4) 2760 W, and (5) 3000 W.

As shown in Figure 8, the number of pores initially increases and then decreases during the underpenetrated state (Y-type), and gradually increases again upon achieving complete penetration (X-type). The stability of the keyhole significantly influences the formation of pores. During the initial stage of welding, an increase in laser power can lead to the formation of bubbles that are likely to be captured by the solidifying front and form pores due to the potential instability of the keyhole [18]. However, as the laser power continues to increase, the keyhole becomes more stable, thereby reducing the formation of pores. In the welding temperature field, upon complete penetration, cooling initiates at the bottom of the weld seam and then progresses upward. During the laser welding process, the outward ejection of metal vapor from within the keyhole generates a vapor vortex at the keyhole opening, drawing argon into the bottom of the keyhole. As the keyhole advances, argon enters the molten pool in the form of bubbles. If the upward floatation rate of the bubbles is lower than the cooling rate of the molten pool, the bubbles will be retained at the bottom of the weld seam, leading to the formation of pores [19].

As illustrated in Figure 7, with the increase in laser power, the area of the HAZ (heat-affected zone) gradually expands, and the boundary between the HAZ and the BM gradually becomes less distinct.

Furthermore, in the vertical direction of the weld zone, inclined columnar grains can be observed. This phenomenon is attributed to the significant temperature gradient between the upper and lower regions of the weld during the welding and cooling processes [20].

### 3.2. Effect of Laser Power on Microstructure

**Figure 9:**
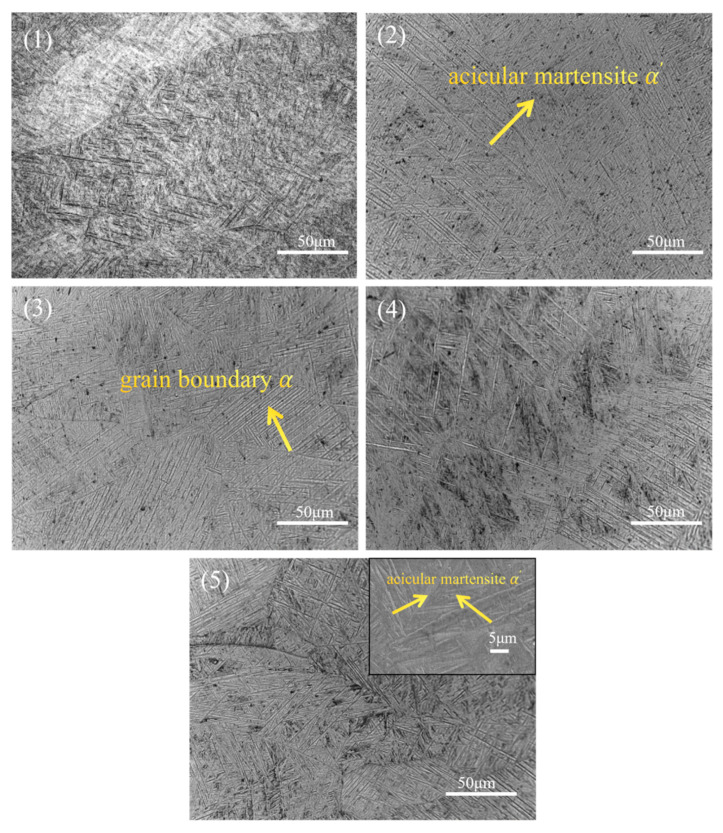
Effect of laser power on the microstructures of the WZ: (1) 2400 W, (2) 2520 W, (3) 2640 W, (4) 2760 W, and (5) 3000 W.

Figure 9 illustrates the microstructural morphology of the WZ (weld zone). It is evident that this region contains a significant amount of acicular martensite α′ phase precipitated from β grains. During the welding process, a pronounced temperature gradient in the WZ causes the β grains to grow rapidly along the temperature gradient direction, ultimately forming columnar β grains. Upon cooling, these β grains undergo solid-state phase transformation, resulting in the precipitation of a substantial quantity of acicular martensite α′ phase and a minor amount of residual high-temperature β phase. As laser power increases, both the aspect ratio and density of the acicular martensite α′ phase further increase. When the laser power is further increased, structures exhibiting characteristic basketweave morphological features may emerge.

**Figure 10:**
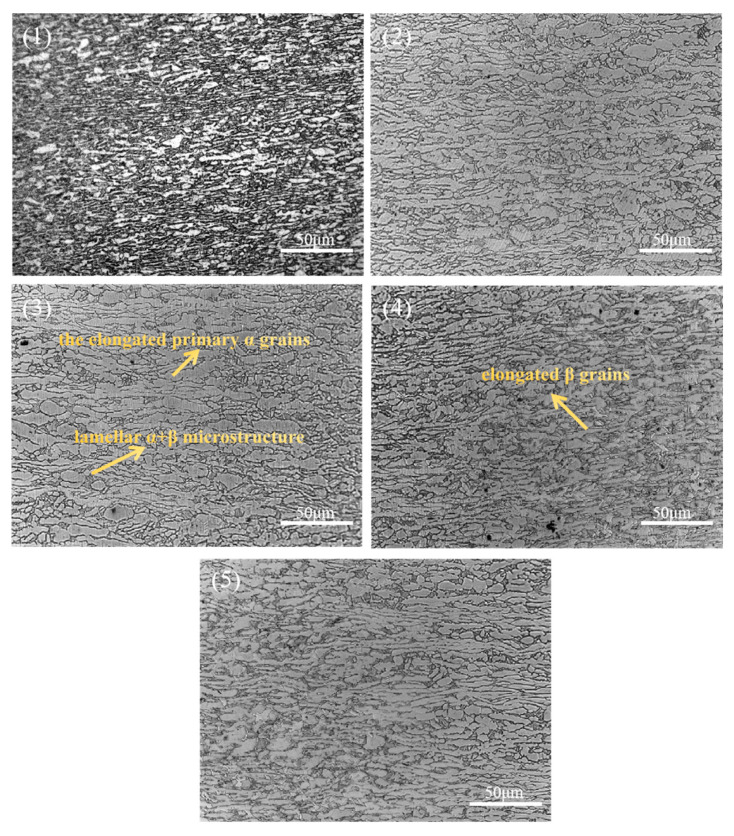
Effect of laser power on the microstructures of the HAZ: (1) 2400 W, (2) 2520 W, (3) 2640 W, (4) 2760 W, and (5) 3000 W.

Figure 10 illustrates the microstructural morphology of the HAZ (heat-affected zone), characterized by a distinct boundary line for organizational changes. At relatively low laser power, the temperature in this region is insufficient to transform primary α grains into β grains; however, the primary α grains are elongated along the temperature gradient direction, resembling columnar grains. As laser power increases, the β grains near the weld seam side exhibit an internal structure rich in martensitic α phase, while the opposite side consists of elongated primary α grains. However, the β grain size in this region is significantly smaller compared to that in the WZ. This phenomenon can be attributed to the faster cooling rate leading to finer grain formation. Within the heat-affected zone (HAZ), β grains located farther from the weld core and closer to the base material experience a steeper thermal gradient and higher cooling rate, thereby promoting the formation of finer β grains.

**Figure 11:**
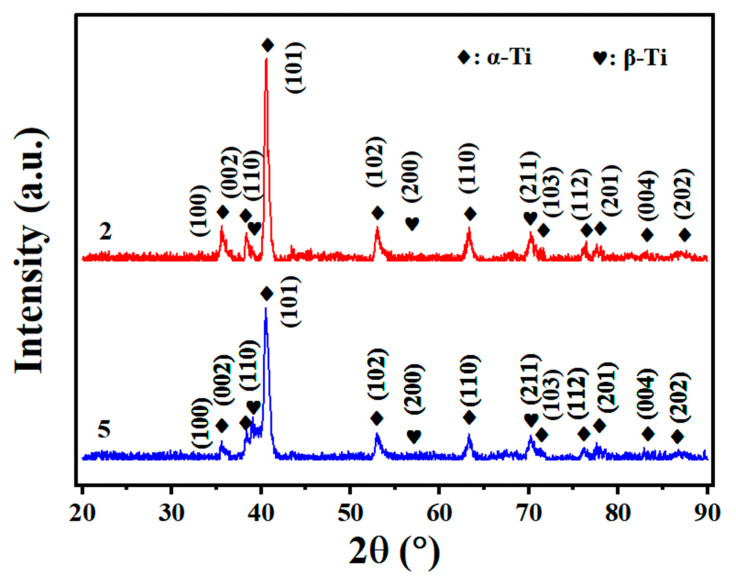
XRD phase calibration: (2) 2520 W and (5) 3000 W.

From Figure 11, it is evident that α-Ti constitutes the largest proportion in the titanium alloy welding joint composition, whereas β-Ti exhibits a relatively minor presence. The primary characteristic peak of α-Ti, which aligns closely with the peak position of α′-Ti, is prominently observed at the (1 0 1) peak around 40.7°. Due to its lower content, the main characteristic peak of β-Ti at the (1 1 0) peak around 38.5° becomes increasingly apparent as laser power increases. This might be due to the preferred orientation of the β phase (texture formed in a certain direction as a result of welding).

Given that Ti80 titanium alloy is a near-α type alloy with inherently low β phase content, rapid cooling following laser welding leads to the transformation of most β phase into acicular α′ phase. Consequently, in the XRD spectrum, the intensity of the third major characteristic peak of the β phase at approximately 56° (2 0 0) is significantly diminished.

**Figure 12:**
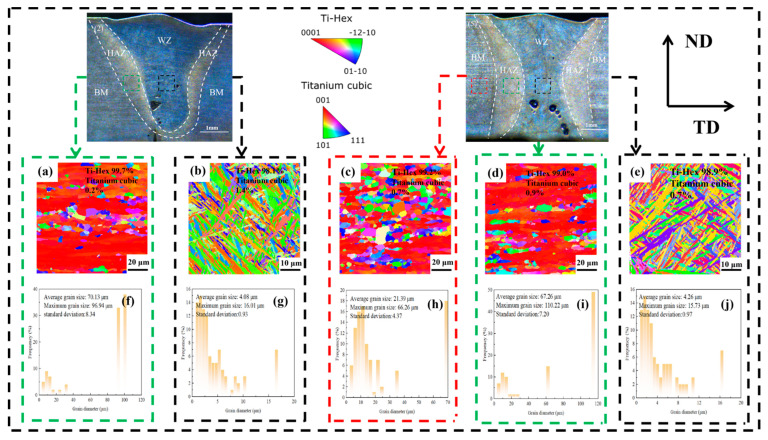
The EBSD maps and grain size of sample 2 and sample 5: (2) 2520 W and (5) 3000 W; (**a**) and (**b**), respectively, represent the HAZ and WZ of sample 2, while (**c**), (**d**) and (**e**), respectively, represent the BM, HAZ, and WZ of sample 5; (**f**,**g**) represent the grain sizes in the HAZ and WZ of sample 2, respectively, while (**h**–**j**) correspond to the grain sizes in the BM, HAZ, and WZ regions of sample 5, respectively.

It can be seen from Figure 12 that the microstructure in the HAZ area of sample 2 is not uniform, including both elongated columnar grains and equiaxed grains. This microstructure distribution is due to the relatively low heat input during welding, which causes some areas of the HAZ to experience a slower cooling rate, resulting in the formation of columnar grains along the temperature gradient direction, while in other areas, local recrystallization occurs, forming equiaxed grains. The grain morphology in the WZ of sample 2 is acicular α′, and even some form a basket-like structure, which is typical of weld microstructure. This is because the rapid cooling characteristics of the weld zone during the welding of sample 2 cause the β phase to decompose into acicular martensitic α′ phase. The grains in the BM area of sample 5 are mainly equiaxed, indicating that the BM microstructure has undergone uniform heat treatment. The grain morphology in the HAZ and WZ of sample 5 is similar to that of sample 2 in the HAZ and WZ. This also indicates that despite the different laser welding powers, the temperature gradient in the HAZ and the rapid cooling in the WZ both lead to similar grain evolution processes.

The Ti-Hex phase is the α phase with an hcp structure (it also contains acicular martensite α′ phase), and the Ti-cubic phase is the β phase with a bcc structure. It can also be seen from the above figure that the main phase of all samples is the Ti-Hex phase. Under constant other process parameters, as laser power increases, the β phase content in the weld zone exhibits a slight decreasing trend, while the α phase content correspondingly shows a marginal increase. This also indicates that the increase in welding heat input results in prolonged exposure to high temperatures and a reduced cooling rate, thereby promoting grain growth in various regions and facilitating the transformation of the β phase in the weld zone into acicular α′ phase.

Figure 12 shows the average grain size of the HAZ of sample 2 is 70.13 μm, and the maximum grain size is 96.94 μm; the average grain size of the HAZ of sample 5 is 67.26 μm, and the maximum grain size is 110.22 μm. The grains in the HAZ are relatively large, mainly due to the welding heat input and thermal cycle causing some areas to stay at high temperatures for a long time, thereby promoting the growth of grains along the temperature gradient direction. The average grain sizes of the WZ of sample 2 and sample 5 are 4.08 μm and 4.26 μm, respectively, and the maximum grain sizes are 16.01 μm and 15.73 μm, respectively, which are significantly smaller than those in the HAZ. This is because the cooling rate in the weld zone is faster, causing the β phase to decompose and form fine acicular martensitic α′ phase.

**Figure 13:**
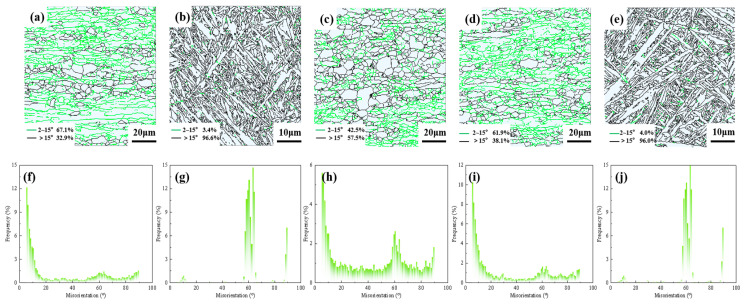
The grain boundary and grain boundary angle distribution diagrams of sample 2 and sample 5: (2) 2520 W and (5) 3000 W; (**a**) and (**b**), respectively, represent the grain boundary distribution maps of the HAZ and WZ of sample 2, while (**c**), (**d**), and (**e**), respectively, represent the grain boundary distribution maps of the BM, HAZ, and WZ of sample 5; (**f**) and (**g**), respectively, represent the grain boundary angle distribution maps of the HAZ and WZ of sample 2; and (**h**), (**i**), and (**j**), respectively, represent the grain boundary angle distribution maps of the BM, HAZ, and WZ of sample 5.

As shown in the above Figure 13, the green lines between 2° and 15° represent Low-Angle Grain Boundaries (LAGBs), while the black lines greater than 15° represent High-Angle Grain Boundaries (HAGBs). In the HAZ of sample 2, LAGBs account for 67.1%, and HAGBs account for 32.9%. In the HAZ of sample 5, LAGBs account for 61.9%, and HAGBs account for 38.1%. In the BM region, LAGBs account for 42.5%, and HAGBs account for 57.5%. The relatively high proportion of LAGBs indicates that the HAZ has undergone partial dynamic recrystallization and retained a large amount of deformed substructure. The proportion of HAGBs in samples 2 and 5 reaches 96.6% and 96%, respectively, which is much higher than that in the HAZ. This suggests that martensite variants with distinct crystallographic orientations have formed in the WZ, where inter-variant misorientation angles predominantly cluster at 60° and 90° [21]. Consequently, the HAGB fraction in the weld region consistently exceeds 95%.

It can also be observed that with the increase in laser power, the HAGB fraction in the WZ exhibited a moderate reduction, decreasing from 96.6% to 96%. However, in the HAZ, the proportion of HAGBs gradually increases while that of LAGBs gradually decreases. This is the combined effect of heat input and thermal cycle, which leads to an increase in the degree of dynamic recrystallization and ultimately increases the proportion of HAGBs.

The recrystallized grain proportion in the HAZ of sample 2 is 16.3%, the substructure grain proportion is 80.6%, and the deformed grain proportion is 3.1%. For sample 5, the recrystallized grain proportion in the HAZ is 21.5%, the substructure grain proportion is 75%, and the deformed grain proportion is 3.5%. Increasing the laser power slightly raises the recrystallization ratio in the HAZ, which might be due to the higher thermal input providing a driving force for recrystallization in this zone. However, the substructure grain proportion in the HAZ decreases when the laser power is increased, possibly resulting in a smaller geometric density of dislocations in this area. In addition, the recrystallized grain fraction in the base material is 42.4%. The recrystallized grain proportions in the WZ of samples 2 and 5 are 34.2% and 32.2%, respectively, significantly higher than those in the HAZ, indicating that the increase in laser power promotes recrystallization in the HAZ while suppressing it in the WZ. Consequently, dynamic recovery may dominate in the WZ [22,23].

The KAM of the HAZ of sample 2 and sample 5 was 0.41° and 0.43°, respectively, while the KAM of the WZ of sample 2 and sample 5 was 0.32° and 0.31°, respectively. It can be seen that the KAM of the HAZ is higher than that of the WZ and the KAM is mostly concentrated on LAGBs, which indicates that the higher proportion of LAGBs in the HAZ leads to a greater local misorientation.

When the laser power was increased, the proportion of LAGBs in the HAZ of sample 2 was higher than that of sample 5. However, the KAM of the HAZ of sample 2 was lower than that of sample 5. Apart from the primary factor of the proportion of LAGBs, it might also be due to the fact that the average grain size of the HAZ of sample 2 was marginally larger than that of sample 5. The dislocation sliding is influenced by the multiple grain boundaries within the material [24,25,26], and the Hall–Petch relationship can also predict that the stress of polycrystalline materials is inversely proportional to the square root of the average grain size [27,28]. That is, the more grain boundaries per unit volume of the material, the greater the hardness. This implies that the smaller the average grain size, the larger the KAM in that area. The same principle applies to the WZ. Hence, it can be deduced that the magnitude of KAM is not only mainly affected by the proportion of LAGBs, but also jointly influenced by factors such as the grain size, the extent of recrystallization, dynamic recovery, and others within that region.

**Figure 14:**
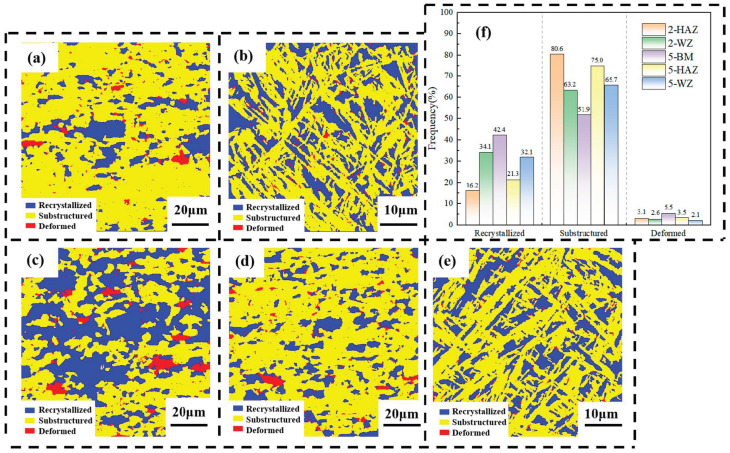
Recrystallization distribution maps of sample 2 and sample 5: (2) 2520 W and (5) 3000 W; (**a**) and (**b**), respectively, represent grain distribution map of the HAZ and WZ of sample 2; (**c**), (**d**), and (**e**), respectively, represent grain distribution map of the BM, HAZ, and WZ of sample 5; (**f**) shows the percentage statistics of recrystallized grains, substructure grains, and deformed grains of samples 2 and 5.

**Figure 15:**
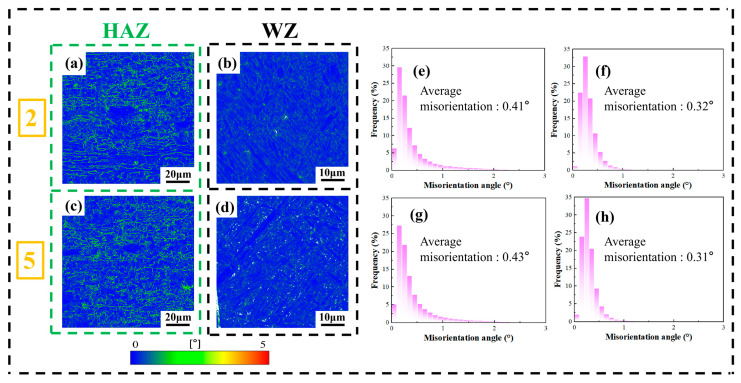
The Kernel Average Misorientation (KAM) maps of sample 2 and sample 5: (2) 2520 W and (5) 3000 W; (**a**) and (**b**), respectively, represent the KAM distribution map of the HAZ and WZ of sample 2; (**c**) and (**d**), respectively, represent the KAM distribution map of HAZ and WZ of sample 5; (**e**) and (**f**), respectively, represent the statistical chart of the KAM angles of HAZ and WZ for sample 2; (**g**) and (**h**), respectively, represent the statistical chart of the KAM angles of HAZ and WZ for sample 5.

**Figure 16:**
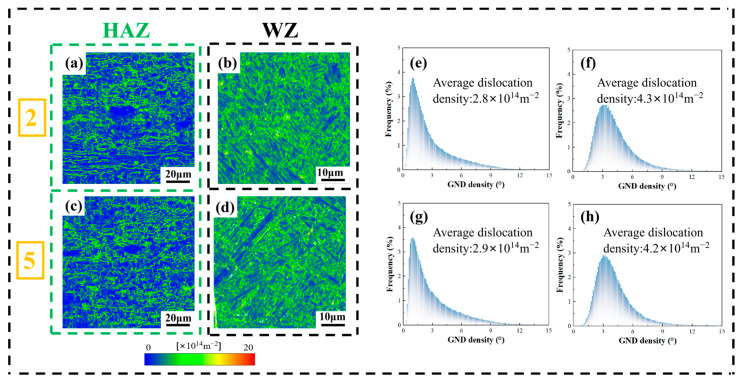
The Geometrically Necessary Dislocations (GND) distribution map and statistical graph of sample 2 and sample 5: (2) 2520 W and (5) 3000 W; (**a**) and (**b**), respectively, represent the GND distribution map of the HAZ and WZ of the sample 2; (**c**) and (**d**), respectively, represent the GND distribution map of the HAZ and WZ of the sample 5; (**e**) and (**f**), respectively, represent the GND statistical graph of the HAZ and WZ for sample 2; (**g**) and (**h**), respectively**,** represent the GND statistical graph of the HAZ and WZ for sample 5.

It can be seen from the above figure that the average GND density of the HAZ of sample 2 and sample 5 is 4.26 × $10^{14}{\;m}^{-2}$ and 4.35 × $10^{14}{\;m}^{-2}$, respectively, while the average GND density of the WZ of sample 2 and sample 5 is 6.4 × $10^{14}{\;m}^{-2}$ and 6.34 × $10^{14\;}m^{-2}$, respectively. From Figure 14, it can be seen that although the average KAM of the WZ of the two samples is lower than that of the HAZ; it is found from the above figure that the average GND density of the WZ of the two samples is higher than that of the HAZ, which is rather abnormal. From Figure 14, Figure 15 and Figure 16, it can be seen that this may be due to the fact that the proportion of small-angle grain boundaries in the HAZ is higher, and the recrystallization degree in this area is lower, with more substructures, resulting in larger local strain and thus larger KAM. While the proportion of HAGBs in the WZ is higher, and the recrystallization degree in this area is higher, which releases part of the local stress, so the KAM is lower. Although the WZ reduces part of the dislocation density after recrystallization, due to the rapid cooling after laser welding, the β phase in the WZ transforms into acicular martensite α′ phase (the microstructure contains high-angle martensite variants), which is a non-diffusive shear and generates a large number of dislocation densities at the grain boundaries. Therefore, the GND in the WZ is larger than that in the HAZ.

KAM is used to assess the uniformity of deformation in the welding area, and it indirectly regulates the macroscopic mechanical properties. The density and distribution of GND directly determine the dislocation strengthening effect and stress concentration. It can be inferred that the hardness and local stress concentration in the WZ are higher than those in the HAZ.

In addition, when the laser power is increased, due to the combined influence of multiple factors, the KAM in the HAZ slightly increases while that in the WZ slightly decreases. This also indicates that the local plastic deformation is relatively uniform before and after welding. Moreover, when the laser power is increased, the GND density in the HAZ slightly increases while that in the WZ slightly decreases, which is consistent with the trend of KAM changes.

### 3.3. Effect of Laser Power on the Vickers Hardness and Tensile Testing

**Figure 17:**
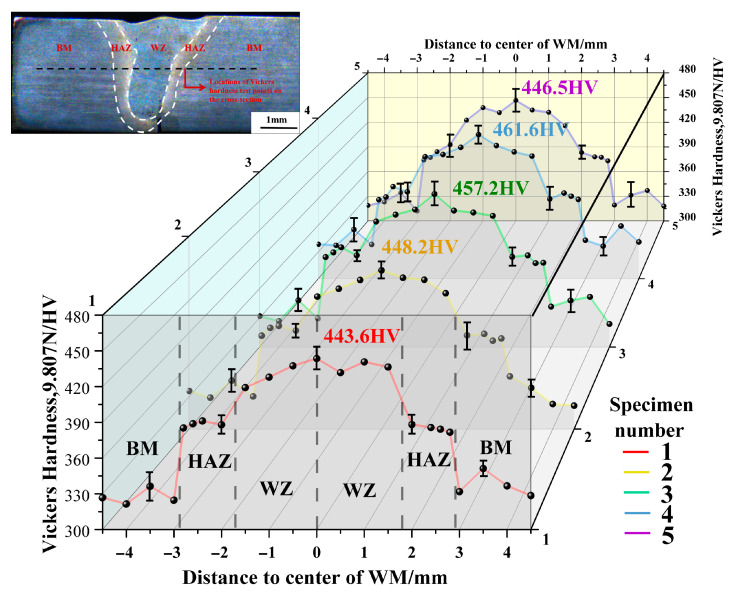
Effect of laser power on the Vickers hardness distribution diagram in the middle of the weld cross-section: (1) 2400 W, (2) 2520 W, (3) 2640 W, (4) 2760 W, and (5) 3000 W.

Figure 17 is the Vickers hardness distribution diagram in the middle of the weld cross-section. The Vickers hardness value of each point is the average of three measurements. As shown in the figure, the average Vickers hardness of the Ti80 BM region ranges from 320 HV to 360 HV, while that of the WZ ranges from 420 HV to 460 HV. It can be seen from the above figure that the Vickers hardness at the center of the weld seam first increases and then slightly decreases. Initially, with increasing laser power, a substantial amount of acicular martensit α′ phase formed in the WZ, and a basketweave structure may also have developed, contributing to an increase in hardness. Subsequently, with the further increase in laser power, the grain size in the WZ slightly increases and the GND density decreases, which in turn leads to a decrease in hardness.

**Figure 18:**
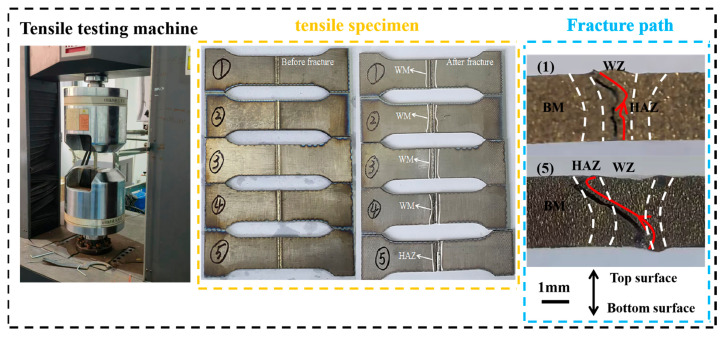
Before and after the fracture of the tensile specimen.

Based on the data presented in Figure 18 and Table 4, tensile specimens 1 to 4 fractured within the WZ, while specimen 5 fractured in the HAZ. As laser power increased, the tensile strength of all specimens exhibited a consistent upward trend; however, no clear pattern was observed in the tensile elongation. Samples 1 to 3 exhibit incomplete fusion penetration. Their microstructure and associated mechanical properties differ significantly from those of samples 4 and 5. These differences may result in localized stress concentrations, thereby affecting tensile strength and elongation [29,30,31]. In contrast, specimens 4 and 5 were fully penetrated. The fracture of specimen 4 in the WZ region can be attributed to the increased formation of acicular α′ grains and fewer equiaxed α grains following the phase transformation of β grains, resulting in diminished plasticity. For specimen 5, the higher laser power introduced greater heat input, leading to an increase in the size of β columnar grains and a higher density of acicular α′ grains in the HAZ region, which consequently reduced the plasticity of this area [32].

**Figure 19:**
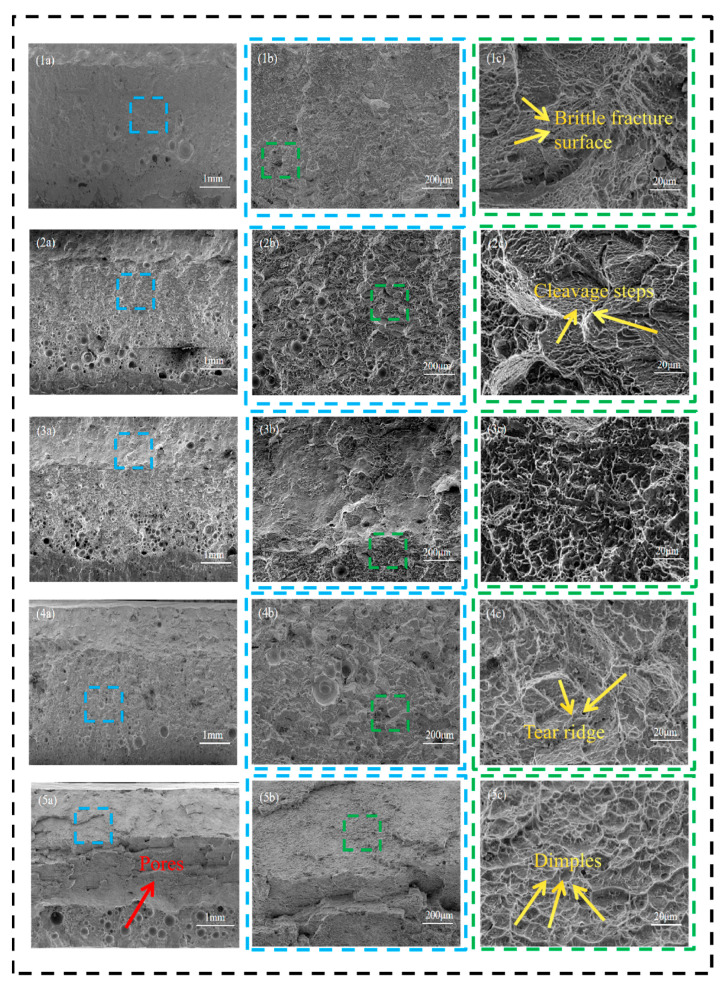
Effect of laser power on the microscopic morphology of tensile fracture: (**1a**–**1c**) 2400 W; (**2a**–**2c**) 2520 W; (**3a**–**3c**) 2640 W; (**4a**–**4c**) 2760 W; (**5a**–**5c**) 3000 W.

Figure 19 illustrates the microscopic morphologies of tensile fracture surfaces for specimens (1) to (3) at 2400 W, 2520 W, and 2640 W (incomplete penetration), (4) at 2760 W (just complete penetration), and (5) at 3000 W (complete penetration). According to Figure 18, when the laser power is relatively low, the fracture surface of specimen (1) to (3) exhibit a smooth appearance with step-like features, but small and shallow ductile dimples are observed at the grain boundaries. The data from Figure 17 and Table 4 indicate that this specimen undergoes brittle fracture, suggesting that even in brittle fracture, localized plastic deformation can occur, forming small dimple-like structures. Specimen (4) shows limited plastic deformation characteristics, with typical coarse tear ridges appearing in the central region. Based on the combined analysis of Figure 18 and Table 4, this specimen demonstrates mixed fracture behavior. Specimen (5) exhibits significant plastic deformation, characterized by a large number of equiaxed ductile dimples. The integrated analysis of Figure 18 and Table 4 confirms that this specimen conforms to the characteristics of ductile fracture.

**Figure 20:**
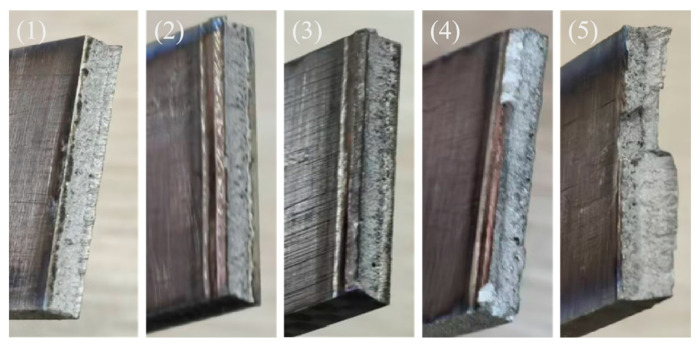
Effect of laser power on the macroscopic morphology of tensile fracture: (1) 2400 W, (2) 2520 W, (3) 2640 W, (4) 2760 W, and (5) 3000 W.

Figure 20 illustrates the macroscopic morphologies of tensile fracture surfaces for specimens. As can be observed from the above Figure 19, the fracture surfaces of tensile specimens 1 to 3 are relatively smooth, exhibiting no evident plastic deformation features. Localized traces are present on the upper portion of specimen 4, whereas specimen 5 displays distinct signs of plastic deformation in the upper region, accompanied by pore clustering in the lower region.

Overall, the fracture mechanism of the tensile specimen of this laser-welded joint can be mainly divided into two types. (1) At low power (2400–2760 W), the WZ is dominated by fine grains and high defects (mainly including incomplete penetration and the entrainment of atmospheric CO_2_ and possible trace organic contamination from the fixture. This local carbon enrichment consequently facilitates the formation of brittle inclusions, including both Ti-C and Ti-C-O compounds in the weld bottom), while the HAZ is relatively narrow at this time, with smaller grain size and no significant degradation of HAZ microstructure and properties. At this time, the tensile specimen fractures start and end in the WZ. (2) At high power (2760–3000 W), the HAZ exhibits an expanded area, coarser grain structure, and localized microstructural softening, accompanied by an increased density of GNDs. Although the WZ demonstrates high hardness, the presence of numerous pores at the bottom promotes crack initiation in this region. Consequently, fracture begins at the bottom of the WZ and progressively propagates, ultimately terminating within the HAZ. Although the average grain size in the HAZ of sample 5 (67.26 µm) is smaller than that of sample 2 (70.13 µm), the degree of recrystallization in the HAZ of sample 5 is greater. Consequently, the local softening in the HAZ of sample 5 (HAZ recrystallization ratio: 21.3%) is more pronounced, leading to the final fracture of its tensile specimen occurring within the HAZ, whereas the tensile specimen of sample 2 (HAZ recrystallization ratio: 16.2%) fractures in the WZ. This behavior results from the interplay of multiple influencing factors.

### 3.4. The Simulation Results of the Temperature Field

**Figure 21:**
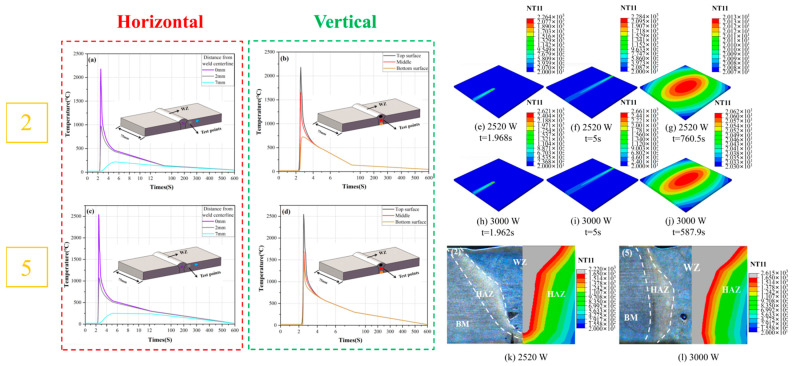
Schematic of central weld cross-section and welding thermal field: the power of the simulated laser heat source utilized in experiments (**a**,**b**,**e**–**g**,**k**) are uniformly set at 2520 W, and the power of the simulated laser heat source utilized in experiments (**c**,**d**,**h**–**j**,**l**) are uniformly set at 3000 W.

As shown in Figure 21, the laser welding process is completed within 5 s, and the plate temperature returns to ambient temperature approximately 10 min thereafter. During the laser welding process, a pronounced temperature gradient is observed, with the peak temperature distributed along the weld seam. When a 2520 W simulated laser heat source is used, the maximum temperature during welding reaches approximately 2284 °C; when a 3000 W simulated laser heat source is employed, the maximum temperature increases to approximately 2661 °C. Both of these peak temperatures are significantly lower than the boiling point of titanium alloy. Consequently, the weld seam surface will be free from significant defects such as metal spatter and porosity. The experimental results demonstrate that as the laser power increases, the maximum temperature during the welding process also rises.

In the horizontal direction, under simulated heat source conditions of 2520 W and 3000 W, the temperature in the weld centerline exceeded 2000 °C, significantly surpassing the melting point of Ti80 titanium alloy (approximately 1650 °C). In contrast, within the HAZ, the temperature reached approximately 1000 °C, which is below the melting point of Ti80 titanium alloy but above the critical temperature for α/β phase transformation (from 900 °C to 1000 °C) [33,34]. Consequently, the α/β phase transformation in the HAZ was incomplete, resulting in a limited amount of β phase and fewer acicular α′ phases precipitating during cooling. In the vertical direction, under the 2520 W simulated heat source condition, the bottom temperature of the plate was only around 750 °C to 800 °C, well below the melting point of Ti80 titanium alloy, indicating that the plate was not fully penetrated at this laser power level. This finding aligns with the results of the previously described laser welding experiments conducted at a laser power of 2520 W.

### 3.5. The Simulation Results of the Stress Field

**Figure 22:**
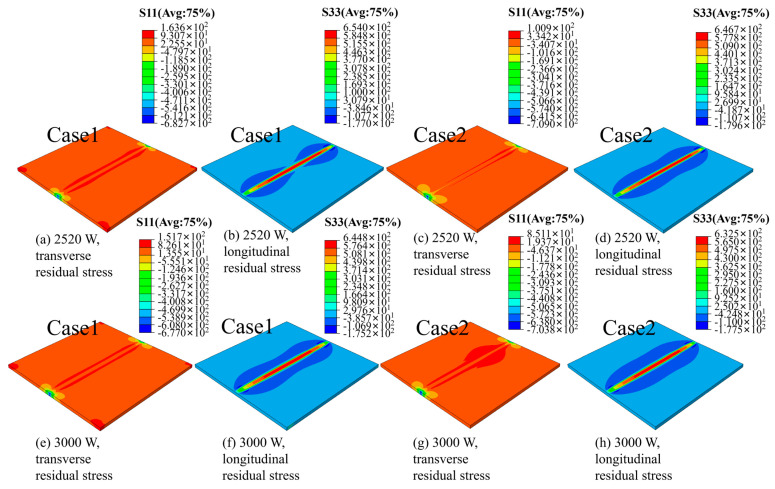
The impact of different fixture approaches and laser power on the distribution of transverse and longitudinal residual stresses: The power of the simulated laser heat source utilized in experiments (**a**–**d**) are uniformly set at 2520 W, and the power of the simulated laser heat source utilized in experiments (**e**–**h**) are uniformly set at 3000 W.

As shown in Figure 22, when the fixture setting mode is case 1, both the transverse and longitudinal residual stresses of the plate are marginally higher compared to those in fixture setting mode case 2. This suggests that the estimated residual stress field under fixture setting mode case 2 is underestimated. In fixture setting mode case 1, a pronounced transverse residual stress concentration is observed around the edges of the plate. Additionally, the longitudinal residual stress significantly exceeds the transverse residual stress. After increasing the laser power, both the longitudinal and transverse residual stresses exhibit a slight reduction. This phenomenon can be attributed to the transition of the weld from incomplete penetration to complete penetration, which mitigates stress concentration.

After laser welding, significant residual stresses are observed along both the weld seam direction and the perpendicular direction within the welded joint, with the transverse residual stress being relatively smaller. This phenomenon is attributed to the predominant application of laser energy along the welding direction, leading to pronounced expansion and deformation of the longitudinal structure. In contrast, the transverse residual stress arises from the expansion and deformation of the cross-sectional structure in the welding direction. Experimental measurements indicate that both types of residual stresses exhibit an asymmetrical double-peak distribution [35,36].

**Figure 23:**
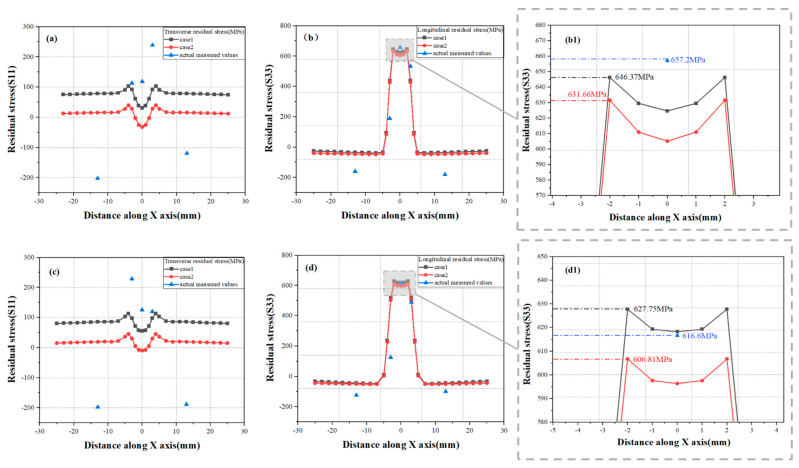
The distribution characteristics of longitudinal and transverse residual stresses: the power of the simulated laser heat source utilized in experiments (**a**,**b**,**b1**) are uniformly set at 2520 W, and the power of the simulated laser heat source utilized in experiments (**c**,**d,d1**) are uniformly set at 3000 W.

Figure 23 illustrates the distribution characteristics of transverse and longitudinal residual stresses in the plate under laser-simulated heat sources at 2520 W and 3000 W. As shown in the figure, in both scenarios, the longitudinal residual stress values are higher and have a more significant impact on the weld joint of the plate. Additionally, changes in fixture configuration notably influence the transverse residual stress, particularly near the WZ and HAZ, where the predicted residual stress values using the case1 fixture are closer to the actual measured values.

Specifically, under condition (1) at 2520 W, the maximum predicted longitudinal residual stress in the WZ by the case1 fixture was 646.37 MPa, while the actual measured value at the weld center was 657.2 MPa, resulting in a relative error of only 1.65%. Under condition (2) at 3000 W, the maximum predicted longitudinal residual stress in the WZ by the case1 fixture was 627.75 MPa, while the actual measured value at the weld center was 616.6 MPa, leading to a relative error of 1.81%. With increasing laser power, the longitudinal residual stress at the weld center decreases, likely due to incomplete penetration of the titanium alloy plate at 2520 W, which causes stress concentration and results in higher longitudinal residual stress.

The quantification of the transverse residual stress error is (1) at a power level of 2520 W, the measured maximum transverse residual stress at the weld seam was 239.1 MPa, whereas the simulated value was 103.4 MPa, resulting in a relative error of 56.754%; (2) at a power level of 3000 W, the measured maximum transverse residual stress at the weld seam was 228.5 MPa, whereas the simulated value was 112.87 MPa, resulting in a relative error of 50.604%.

The residual stress analysis of titanium alloy plates following laser welding involves a complex coupling of thermodynamics, metallurgy, and mechanics, which makes the achievement of fully accurate prediction and analysis highly challenging. The error sources and sensitivity of the transverse residual stress differ to some extent from those of the longitudinal residual stress. These possible factors have been analyzed: (1) physical influencing factors of welding fixtures: in the actual welding process, the number of constraints and positioning elements provided by the welding fixture is generally greater than that defined in the simulation setup. As indicated by the cited references [36,37], an increase in constraint quantity tends to elevate the transverse residual stress to a certain extent. Consequently, this could be one of the contributing factors to the observed higher transverse residual stress values compared to the simulation results; (2) influencing factors of thermal stress: in the actual welding process, the contact area between the welding fixture and the workpiece is significantly large and cannot be neglected. Consequently, the heat dissipation coefficient in the actual process is generally higher than the value defined in the simulation setup. As a result, the post-welding cooling rate tends to be faster, leading to measured residual stress values that are often slightly higher than those obtained from simulation; (3) the inhomogeneity of material composition distribution in welded plates: during the simulation process, the material composition distribution is assumed to be perfectly uniform. In contrast, in actual welding processes, the material composition distribution of the welded plates typically exhibits a certain degree of non-uniformity, which exerts a significant influence on the post-weld residual stress distribution; (4) influencing factors of martensitic transformation: as indicated by the cited reference [37], during the simulation process, when the phase transformation effect is considered, the maximum transverse residual stress exhibits a variation range of 50% (whereas the longitudinal residual stress does not show such a significant change). Furthermore, when the phase transformation effect is taken into account, the overall transverse deformation of the welded plate remains relatively small. This suggests that a smaller proportion of the transverse residual stress is released through plastic deformation, resulting in a relatively higher amount of stored residual stress.

In conclusion, the simulation and prediction analysis of residual stress after laser welding of titanium alloys is a complex coupling of mechanics, metallurgy, and thermodynamics, and it is extremely difficult to conduct a complete and comprehensive simulation and prediction analysis. During the simulation process, compared with the longitudinal residual stress, the transverse residual stress is more sensitive to the settings of the welding fixture and the phase transformation effect, and is more easily affected by these two factors, thereby influencing the accuracy of the simulation.

## 4. Conclusions

This study employed an experimental-simulation combined approach to reveal the influence of laser power on the microstructure and mechanical properties of Ti80 titanium alloy laser-welded joints. Through multi-scale analysis, the relationships among process parameters, microstructure, and mechanical properties were established and analyzed, providing a reference for laser welding of Ti80 alloy in marine equipment. However, due to the influence of multi-factor synergy/competition, the mechanism of the effect of a single variable on performance still needs to be further analyzed in depth.

- Increasing laser power changes weld morphology from Y-type to X-type and affects pore distribution, with more pores in the lower weld areas. In incompletely penetrated welds, pore numbers rise then fall, while in fully penetrated ones, they increase with power.
- In laser welding of Ti80 titanium alloy, the density of GND in the WZ is slightly higher than that in the HAZ. Increasing laser power not only reduces the recrystallization degree of WZ grains but also decreases GND density in WZ, and the Vickers hardness of the WZ first increases and then decreases. The average Vickers hardness of the WZ is approximately 420 HV to 460 HV, that of the HAZ is about 390 HV to 400 HV, and that of the BM is approximately 320 HV to 360 HV.
- All tensile specimens 1 to 4 started to fracture at the lower part of the WZ and the cracks ended at the upper part of the WZ. However, specimen 5 began to fracture in the HAZ and the crack ended at the upper part of the HAZ. The tensile strength of specimen 5 was 903.12 MPa and the tensile elongation was 10.4%. There are two mechanisms governing the fracture behavior of tensile specimens: 1. At low power levels (2400–2760 W), the WZ is characterized by fine grains and high defect density, while the HAZ maintains relatively stable microstructures and mechanical properties. Under these conditions, although the high proportion of HAGBs in the WZ inhibits crack formation, fracture is primarily attributed to critical defects within the WZ, including incomplete penetration and insufficient weld strength. 2. At high power levels (2760 W–3000 W), the HAZ undergoes significant expansion, leading to grain coarsening and localized softening. Concurrently, the proportion of high-angle grain boundaries in the WZ slightly decreases. Despite the relatively low residual stress in the WZ, pore formation predominantly concentrates at its bottom region, which becomes the critical factor for crack initiation. Consequently, fracture behavior is jointly influenced by both the HAZ and WZ regions.
- In the simulation experiment, the simulation outcomes indicated that under the heat source condition of 2520 W, the temperature at the bottom of the plate was too low, resulting in incomplete weld penetration, which was in accordance with the actual experimental circumstances. In the stress field analysis, the errors between the maximum longitudinal residual stress in the WZ obtained through simulation under two different heat source power conditions and the actual measured longitudinal residual stress at the center of the WZ were 1.65% and 1.81%, respectively. The transverse residual stress is more significantly influenced by the clamping configuration and martensitic phase transformation, resulting in relative errors as high as 56.754% and 50.604%, respectively.

## Figures and Tables

**Table 1:** Chemical composition of Ti80 (wt%).

Al	Nb	Zr	Mo	Si	Fe	C	O	N	H	Ti
6.05	2.68	2.06	1.07	0.012	0.074	0.027	0.107	0.007	0.0013	Bal

**Table 2:** Laser welding parameters.

Specimen Number	Laser Power/kw	Welding Speed/ $\mathrm{mm}\cdot s^{-1}$	Laser Defocusing/mm	Laser Heat Input/mm^−1^	Shielding Gas
1	2.40	30	+3	80	
2	2.52	30	+3	84	
3	2.64	30	+3	88	Ar
4	2.76	30	+3	92	
5	3.00	30	+3	100	

**Table 3:** Inherent properties of Ti80 titanium alloy.

Density $\rho /(\mathrm{kg}\cdot m^{-3})$	Poisson Ratio μ	Latent Heat $T_{3}$$/(j\cdot kg^{-1})$	Solidus Temp $T_{2}/\left(℃\right)$	Liquidus Temp $T_{1}/\left(℃\right)$
4657	0.33	300,000	1650	1716

**Table 4:** Tensile test results of WM under different laser power.

Specimen Number	Laser Power/W	Tensile Strength/ MPa	Tensile Elongation/%	Fracture Position
1	2400	359.06	2.69	WM
2	2520	454.06	1.48	WM
3	2640	508.18	5.13	WM
4	2760	676.41	3.27	WM
5	3000	903.12	10.40	HAZ

## Data Availability

The original contributions presented in this study are included in the article. Further inquiries can be directed to the corresponding authors.

## Abbreviations

WM	Weld metal
WZ	Welding zone
HAZ	Heat affected zone
BM	Base material zone
KAM	The Kernel Average Misorientation
GND	The Geometrically Necessary Dislocations
